# A Rare Complication after Synthetic Meniscus Replacement

**DOI:** 10.5334/jbsr.1601

**Published:** 2018-10-03

**Authors:** Laurence Verhaeghe, Karolien Boeren

**Affiliations:** 1 KU Leuven, BE; 2 AZ Delta, BE

**Keywords:** meniscectomy, postmeniscectomy syndrome, meniscus replacement, complication, NUsurface

## Case

**Figure 1:**
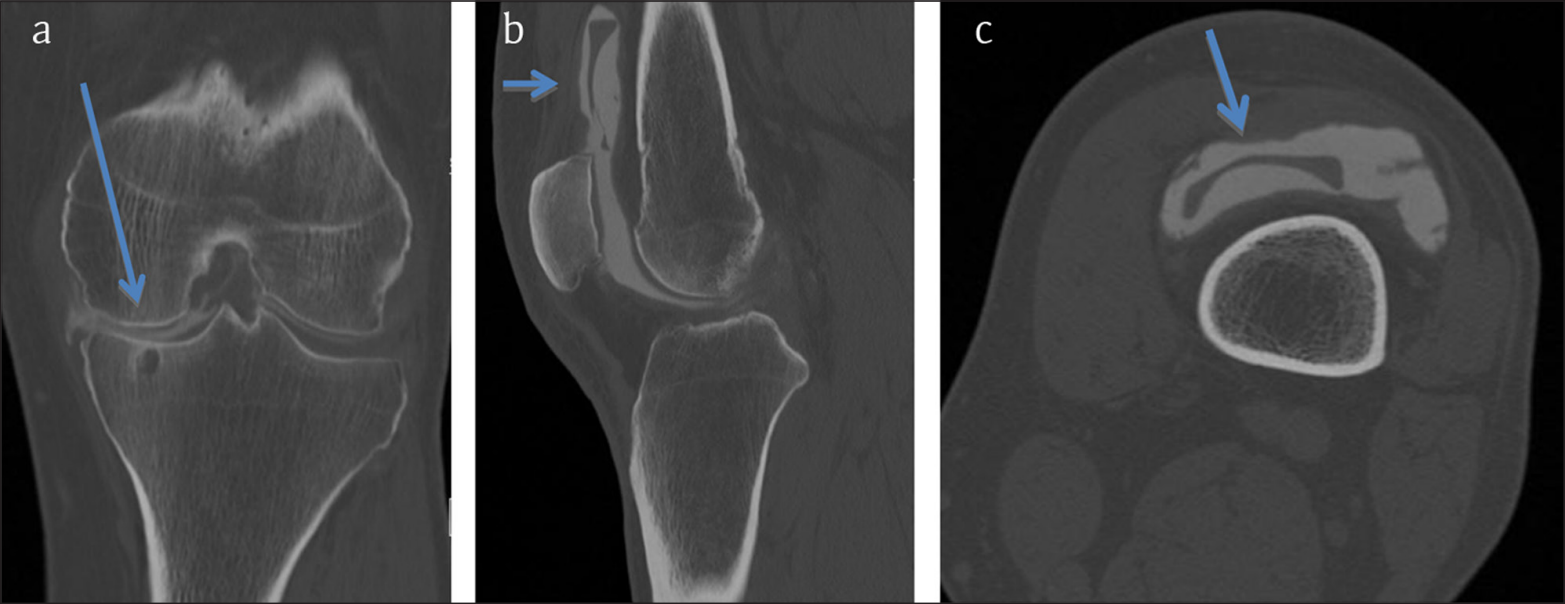
Arthro-CT with coronal **(a)**, sagittal **(b)** and axial **(c)** views.

A 55-year-old male patient presented to the orthopaedic department with the complaint of a left knee blockage. Five years earlier, he underwent a left knee meniscectomy for a posttraumatic medial meniscus tear. Because of persisting pain and swelling of the medial compartment (typical post-meniscectomy syndrome) he underwent synthetic PCU (polycarbonate-urethane) meniscus replacement (type NUsurface^R^), which resulted in regain of full functionality. However, he started experiencing a clicking sensation and sometimes a complete blockage of his left knee a few weeks prior to the consultation. A computed tomography (CT) arthrography was performed. On the coronal view (Figure 1a) there was a complete opacification of the medial femorotibial joint space devoid of any meniscal structure, together with a degenerative tibial subchondral geode (arrow). The sagittal (Figure 1b) and axial (Figure 1c) views showed a hypodense, wedge-shaped structure in the suprapatellar space: the luxated and superiorly migrated synthetic meniscus (arrows).

## Discussion

The menisci are important elements of the knee joint; besides augmenting the articular surface between femur and tibia to reduce the stress on the cartilage, they also function as a shock absorber and contribute to joint stability and joint lubrication.

After knee trauma with meniscal injury, the goal is to preserve as much meniscal tissue as possible. But, because of the centripetal vascularity of the menisci, not all tears can be repaired. Therefore, a partial or total meniscectomy can be inevitable. As a consequence, the cartilage is exposed to higher stress and an accelerated progression to arthritis can be expected.

Since its first introduction in 1989, meniscal allotransplantation has become a valuable therapeutic option and the best treatment available for symptomatic patients post-meniscectomy. However, problems related to graft availability, size matching, cost-effectiveness and disease transmission limit their widespread use [1]. These obstacles led to an extensive search for a synthetic biomaterial that provides the optimal compromise between flexibility and strength, mimicking the human meniscus as much as possible.

NUsurface^R^ is the first synthetic total meniscal substitute used in humans and consists of polyethylene reinforced polycarbonate urethane. In vivo experiments in sheep showed promising results with a short-term chondroprotective effect [1]. Further investigation is needed to show whether or not the same positive effect is seen in long-time follow-up. There are two on-going trials; one to prove the safety and effectiveness of the implant in restoring functionability (SUN-trial) and the other to compare the results of the NUsurface^R^ meniscus implant to non-surgical standard of care (VENUS-trial).

Because of its shape and geometry, the NUsurface^R^ prosthesis doesn’t require any fixation to bone or soft tissue. Loosening, luxation and subsequently migration of the meniscal substitute (as seen in the case above) is therefore a possible complication. Especially because of the increasing clinical implementation of this promising surgical technique, it’s an image that radiologists may encounter more often in the future.
